# Prevention of Immune Nephritis by the Small Molecular Weight Immunomodulator Iguratimod in MRL/lpr Mice

**DOI:** 10.1371/journal.pone.0108273

**Published:** 2014-10-01

**Authors:** Qingran Yan, Fang Du, Xinfang Huang, Qiong Fu, Sheng Chen, Dai Dai, Chunde Bao

**Affiliations:** 1 Department of Rheumatology, Renji Hospital, School of Medicine, Shanghai Jiaotong University, Shanghai Institute of Rheumatology, Shanghai, China; 2 Institute of Health Science, Shanghai Institute for Biological Science, Chinese Academy of Science and Shanghai Jiaotong University School of Medicine, Laboratory of Molecular Rheumatology, Shanghai, China; University Paris Sud, France

## Abstract

**Objective:**

This study was performed to investigate the therapeutic effects of iguratimod in a lupus mouse model.

**Methods:**

Female MRL/lpr mice were treated with iguratimod, vehicle solution or cyclophosphamide. Proteinuria was monitored and kidney injury was blindly scored by a renal pathologist. Serum anti-double-stranded DNA antibodies were monitored by radioimmunoassay. Kidney IgG and CD20 were stained by immunohistochemistry. Splenic lymphocyte phenotypes were analyzed by flow cytometry. BAFF, IL-17A, IL-6, and IL-21 levels in serum and splenic lymphocytes were detected by ELISA or quantitative PCR.

**Results:**

Compared with the vehicle-treated controls, MRL/lpr mice treated with iguratimod showed less protenuria, less acute pathological lesions and no chronic changes in the kidneys. There were significant differences in glomerular injury and vasculitis scores, as well as in the semi-quantitave analysis of immune complex deposition between the two groups. Disease activity markers in sera (anti-dsDNA antibodies and immunoglobulin levels) were reduced and hypocomplementemia was attenuated. Lymphocyte expression of BAFF, IL-6, IL-17A and IL-21 was decreased. The abnormal splenic B220+ T cell and plasma cell populations in MRL/lpr mice were reduced by iguratimod treatment, with recovery of the total B cell population and inhibition of B cell infiltration of the kidney tissue. The dosage of iguratimod used in this study showed no significant cytotoxic effects *in vivo* and no overt side-effects were observed.

**Conclusion:**

Iguratimod ameliorates immune nephritis in MRL/lpr mice via a non-antiproliferative mechanism. Our data suggest a potential therapeutic role of iguratimod in lupus.

## Introduction

Iguratimod (iguratimod, N-[7-[(methanesulfonyl) amino]-4-oxo-6-phenoxy-4H-1-benzopyran-3-yl] formamide) is a small molecular weight immunomodulator. We, and other researchers, have demonstrated the therapeutic effect of iguratimod both in the collagen-induced arthritis (CIA) model [1], [2] and in clinical trials for rheumatoid arthritis (RA). [3]–[5] This agent has been approved for treating RA in several countries over the last 3 years.

During the last two decades, a series of studies have demonstrated multiple immunomodulatory effects of the iguratimod. This agent inhibits nuclear factor-κB activity, [6] blocks IL-17 signaling, [7] stabilizes the lysosome membrane [8] and suppresses inflammatory cytokines [1] both *in vivo* and *in vitro*. Notably, iguratimod reduces immunoglobulin production by acting directly on B cells in both mice and humans, despite having no notable effect on B cell proliferation. [9] We also observed decreased serum anti-type II collagen antibody titers in the CIA model following iguratimod treatment. [1] In clinical trials, serum levels of rheumatoid factor and immunoglobulin were also decreased in RA patients after iguratimod treatment. [4]

Based on these observations, we hypothesized that iguratimod is a plausible candidate for the treatment of another autoimmune disease, systemic lupus erythematosus (SLE), since all of the mechanisms described participate in the development of this disease. [10]–[13]

We tested our hypothesis in the MRL/lpr mouse model of lupus. This strain spontaneously develops an autoimmune syndrome characterized by immune nephritis and autoantibody production, which closely resembles human (SLE). In these mice, Fas-dependent apoptosis is deficient because of a mutation in the *lpr* gene, [14] leading to a rapid acceleration and deterioration of the autoimmune condition driven by genes of the MRL mouse lineage. [15]

Therefore, we treated MRL/lpr mice with iguratimod, vehicle solution or cyclophosphamide. We assessed the effects of iguratimod on immune nephritis, proteinuria, kidney histology and serum markers, as well as its cellular and molecular effects on lymphocytes in MRL/lpr mice.

## Materials and Methods

### Treatment of mice

Iguratimod was kindly provided by Simcere Pharmaceutical Group (Nanjing, China). Female MRL/lpr mice were purchased from the Shanghai Laboratory Animal Center and were housed under specific pathogen-free conditions. All of the experimental protocols involving animals and their care were approved by the Committee on Use of Human & Animal Subjects in Teaching and Research of the Shanghai Jiaotong University School of Medicine, and were carried out in accordance with the regulations of the Department of Health of Shanghai. All surgery was performed under chloral hydrate anesthesia, and all efforts were made to minimize suffering. All mice were sacrificed using cervical dislocation.

To assess the effects on the treatment of nephritis treatment, 8-week old mice were selected at random for oral administration of iguratimod (30 mg/kg d, n = 14) or vehicle solution (1% carboxyl methyl cellulose, CMC solution, n = 15) for 20 weeks before being sacrificed. For analysis of serum immunology and lymphocyte subsets, female MRL/lpr mice (aged 10 weeks) were treated orally with iguratimod (30 mg/kg d, n = 5), vehicle solution (1% CMC solution, n = 5) or with cyclophosphamide (20 mg/kg w, n = 5) intraperitoneally as a positive control. Animals were sacrificed after 8 weeks of treatment.

### Serum and urine analysis

Blood samples were collected every 4 weeks from 7 weeks of age. Serum C3 was detected by ELISA (ICL lab, Portland, OR, USA), anti-double stranded DNA (dsDNA) antibody titers were quantified by radioimmunoassay and serum alanine transaminase (ALT), creatinine and blood cell counts were analyzed by using an autoanalyzer.

To determine kidney injury in MRL/lpr mice, urine samples were collected every 3 weeks from the 7 weeks of age by placing the mice individually in metabolic cages for 24 h. Urine protein concentrations were measured by bicinchoninic acid (BCA) protein assay (Thermo Fisher, Waltham, MA, USA).

For histological analyses, kidneys were fixed in paraformaldehyde, embedded in paraffin, stained with H&E and evaluated by a renal pathologist, who was blinded to the treatments of the mice. Glomerular injury was scored from 0 to 4 for each glomerulus and each sample involved evaluation of 30 glomeruli, within at least three separate parts of one section. The average of these 30 values was calculated as the injury score of an individual sample.

### Immunohistochemical (IHC) staining of kidney samples

Immunohistochemistry for IgG deposition and CD20+ B cell infiltration was performed in paraformaldehyde-fixed kidney sections. After deparaffinizing in xylene, absolute ethanol and 95% ethanol, tissue sections were placed in citrate buffer (pH 6.0) and heated at 99.0°C for 20 minutes. Endogenous peroxidase activity was inhibited by treatment with 0.3% hydrogen peroxide for 15 minutes at room temperature. The tissue sections were blocked in goat serum for 20 minutes at room temperature, incubated overnight at 4°C with goat-anti-mouse polyclonal IgG (ICL Lab) or rabbit-anti-human monoclonal CD20 (Abcam, Cambridge, MA, USA) antibodies. For CD20 staining, sections were incubated for a further 2 hours at room temperature with horseradish peroxidase-labeled goat-anti-rabbit IgG (Abcam) as the secondary detection antibody. Immune reactivity was detected using a DAB kit (Biosciences, San Jose, CA, USA) and then slides were counterstained with hematoxylin. Immunohistochemical staining data were acquired using the OlyVIA system (Olymphus, Southend-on-Sea, UK) and semi-quantitatively analyzed by Image Pro Plus 6.0 (Media Cybernetics, Rochville, MD, USA).

### Enzyme-linked immunosorbent assay (ELISA) for detection of serum immune markers

Mouse serum B cell activation factor (BAFF), C3 and immunoglobulin subtypes were detected with commercial ELISA kits (R&D system, Minneapolis, MN and ICL Lab).

### Real-time quantitative polymerase chain reaction (PCR) analysis of cytokine expression

Total RNA from splenic lymphocytes was isolated with TRIzol reagent (Life Technologies, Grand Island, NY, USA) and reverse-transcribed using Sensiscript RT Kit (Life Technologies). mRNA expression of mouse GAPDH, BAFF, IL-6, IL-21 and IL-17 was determined by real-time PCR using SYBR Green Master Mix (Life Technologies) using the following primers: GAPDH (forward) 5′-AGGTCGGTGTGAACGGATTTG-3′, (reverse) 5′-TGTAGACCATGTAGTTGAGGTCA-3′; BAFF (forward) 5′-CCACCGTGCCTCTGTTTTTG-3′, (reverse) 5′-TGCGGAGTGATGGGATCATATC-3′; IL-6 (forward) 5′-CTGCAAGAGACTTCCATCCAG-3′, (reverse) 5′- AGTGGTATAGACAGGTCTGTTGG-3′; IL-17A (forward) 5′-TCAGCGTGTCCAAACACTGAG-3′, (reverse) 5′-GACTTTGAGGTTGACCTTCACAT-3′IL-21 (forward) 5′-GGGGACAGTGGCCCATAAATC-3′, (reverse) 5′-TGGAGCTGATAGAAGTTCAGGA-3′. Thermocycler conditions included an initial incubation at 95°C for 15 s. This was followed by a two-step PCR program: 95°C for 5 s and 60°C for 30 s for 40 cycles. Each reaction was performed in triplicate. Data were collected and quantitatively analyzed on an ABI PRISM 7900 sequence detection system (Applied Biosystems, Grand Island, NY, USA). The GAPDH gene was used as an endogenous control. The amount of gene expression was then calculated as the difference in cycle threshold (ΔCT) between the CT value of the target gene and β-actin. ΔΔCT is the difference between the ΔCT values of the test sample and the control. Relative expression of target genes was calculated as 2-ΔΔ*CT*.

### Flow cytometric analysis of mouse splenic lymphocyte subsets

Mouse spleen cells were isolated by disruption of spleens. Erythrocytes were lysed by hypotonic shock in a potassium acetate solution. Cells were then incubated for 30 min at 4°C with the following antibodies: PercpCy5.5-CD19, PE-CD138 (BD Biosciences) and APC-B220 (Miltenyi, **Teterow**, Germany). Spleen cell suspension data were acquired on a FACSCalibur flow cytometer (BD Biosciences) and analyzed using FlowJo software (Tree Star, Ashland, OR, USA).

### Flow cytometric analysis of mouse splenic lymphocyte apoptosis *in vivo*

Splenic lymphocyte were isolated as described previously. The early and late apoptotic cells were double-stained with annexin V and propidium iodide (PI) using a commercial kit (BD Pharmingen).

### Statistical analysis

Statistical significance of all quantitative data was analyzed with a two-tailed, non-paired Student's *t-*test; semi-quantitative data (including integrity optical density value and kidney injury score), relative cytokine expression levels and lymphocyte proportions were analyzed with a non-parametric Mann-Whitney U-test (SAS Institute, Inc., Cary, NC, USA). Data are shown as mean ±SEM when Student's *t-*test was applied or median with quartile range (QR) when non-parametric tests were applied. An asterisk (*) denotes *p*<0.05, ** denotes *p*<0.01, and *** denotes *p*<0.001.

## Results

### Iguratimod attenuated renal injury and other lupus-related manifestations in MRL/lpr mice

**Figure 1:**
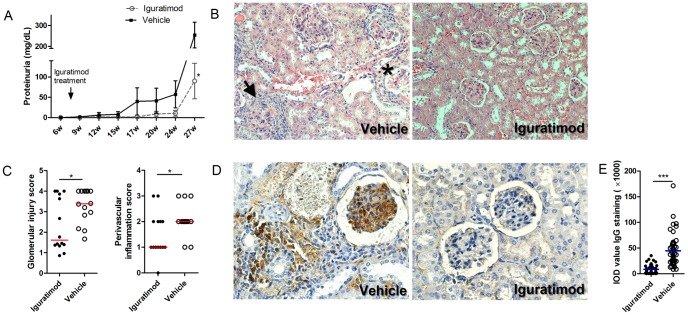
Iguratimod attenuated proteinuria and kidney injury in MRL/lpr mice. (A) Protein concentrations of 24 h-urine from iguratimod treated and control mice. Each dot represents mean ±SEM at the time point. (B) Representative H&E-stained kidney sections from 28 weeks old mice treated with iguratimod or vehicle solution. Control mice develop crescents (arrow), glomerular sclerosis (asterisk), lymphocyte infiltrations and casts in kidneys. Fields are at 200× magnification. (C) Glomerular injury and vasculitis were scored by a renal pathologist blindly. Each dot represents average score of an individual mouse. The horizontal lines represent media. (D) Representative kidney sections stained with IgG after 20-week treatment of iguratimod or vehicle solution. The fields are at 400× magnification. (E) Semiquantitative IOD values of all samples are analyzed (right). Each dot represents mean IOD value of an individual mouse. The horizontal lines represent media. Statistics are calculated by non-paired student's t test (A) or Mann-Whitney U test (C and E). * *p*<0.05, *** *p*<0.001.

Iguratimod therapy remarkably delayed development of proteinuria in MRL/lpr mice. At the age of 24 weeks, iguratimod-treated mice showed only trace amounts of protein in urine, while control mice began to develop notable proteinuria from 15 weeks of age. At the end of the observation, proteinuria level in iguratimod-treated mice was less than 40% of those in the control group (Fig. 1A). In accordance with the effects on proteinuria, iguratimod prevented elevation of serum creatinine in MRL/lpr mice in the late stage of the observation (Fig. S1).

Reflecting the effects on proteinuria, control mice developed large amounts of lymphocyte invasion, creasants, glomerular sclerosis, casts in the tubula and vasculitis in the kidneys; histological assessment scored by a pathologist blindly revealed moderate to severe glomerular damage and signs of perivascular inflammation in all of the control mice (Fig. 1B). In contrast, the kidneys of iguratimod-treated mice showed much milder glomerular damage (median: 1.615, QR 1.360–3.895 vs. median: 3.385, QR 2.665–4.0, *p* = 0.0376) and perivascular inflammation (median: 1, QR 1–2 vs. median: 2, QR 2–2, *p* = 0.0447) (Fig. 1C). Of note, chronic pathological changes, including vascular sclerosis and fibrosis, were present only in control mice, indicating that nephritis progression was restrained by iguratimod.

IgG IHC staining of the kidney tissue revealed marked membranous and subendothelial deposition of IgG immune complexes and occasional staining of capillaries within the glomeruli of vehicle solution-treated mice. In contrast, there was little or no IgG deposition in the glomeruli of mice treated with iguratimod. (Fig 1D) Semi-quantitative analysis of integrity optical density (IOD) scanning confirmed this result (median: 44.73, QR 13.06–132.5 vs. median: 363.2, QR 254.3–587.1, *p*<0.0001) (Fig 1E).

Besides renal involvement, MRL/lpr mice develop other symptoms driven by autoimmunity, including arthritis. [15] In our study, swollen paws were apparent in the control mice at age of 18 weeks, yet iguratimod-treated mice showed only a minimal increase in paw thickness (1.342±0.0084 mm vs. 1.554±0.054 mm, *p* = 0.0012) (Fig S2).

### Iguratimod improved serum markers of nephritis in MRL/lpr mice

**Figure 2:**
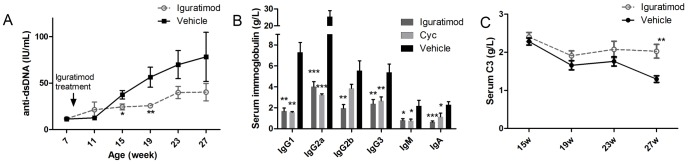
Iguratimod improved serum markers for nephritis activation in MRL/lpr mice. (A) Serum anti-dsDNA antibody levels from iguratimod treated and control mice by radioimmunoassay. Each dot represents mean ±SEM at the time point. (B) Subtype concentrations of serum immunoglobulin after 8-week treatment of iguratimod, Cyc or vehicle solution. Data are represented as mean ±SEM. (C) Serum complement C3 levels of mice with iguratimod or vehicle solution treatment. Each dot represents mean ±SEM at the time point. Statistics are calculated by non-paired student's t test. * *p*<0.05, **p<0.01, *** *p*<0.001.

Autoantibodies are present before proteinuria in MRL/lpr mice [15]. In our study, most mice treated with vehicle solution developed high levels of anti-dsDNA antibodies before 15 weeks of age, indicating disease progression. In iguratimod-treated mice, the antibody level remained stable near baseline level at age of 19 weeks of age, and was approximately half the level found in the control at the end of the study. Of note, iguratimod delayed the appearance of antibodies and decreased antibody titers even when iguratimod-treated mice had higher antibody levels than those observed in the control mice at the beginning of the study. (Fig. 2A).

All serum immunoglobulin isotype concentrations detected, including IgG1, IgG2a, IgG2b, IgG3, IgM and IgA, were strongly decreased after 8 weeks of iguratimod treatment, while the vehicle-treated control mice developed severe hyperglobulinemia. Some of the iguratimod-treated mice showed even lower levels than the cyclophosphamide (Cyc)-treated mice (Fig. 2B).

Iguratimod treatment also remarkably improved hypocomplementemia, which is another serum marker characteristic of lupus nephritis in MRL/lpr mice. [16] The serum complement C3 levels remained stably high following iguratimod treatment, especially in the late stage of the study. In contrast, serum complement C3 levels in control mice decreased from 19 weeks of age to approximately half of the baseline levels at the end of the study. (Fig. 2C)

### Iguratimod affected lymphocyte subsets without overt cytotoxicity

Multiple abnormities in lymphocyte subsets develop in MRL/lpr mice around 10 weeks of age; even earlier than the presence of autoantibodies can be detected. [17] MRL/lpr mice were treated with iguratimod, Cyc, or vehicle solution from 10 weeks of age and spleens were dissected after treatment for 8 weeks. Iguratimod and Cyc both decreased the total splenic lymphocyte number, although the decrease mediated by iguratimod was milder than that of Cyc, and no significant difference was found compared with the vehicle control group (Fig S3)

**Figure 3:**
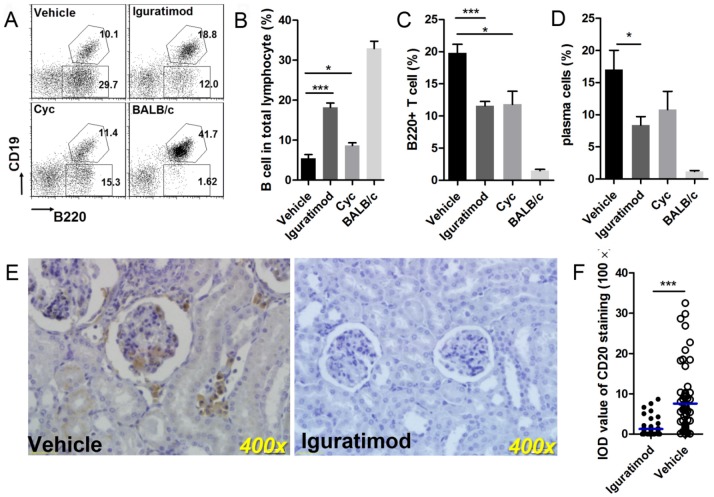
Iguratimod affected lymphocyte subsets. (A) Gating strategy for B220+T cell and total B cell after 8-week treatment. Hexagon gates denote B cell and rectangle gates denote abnormal B220+T cell. (B) Iguratimod and Cyc both recover B cell population in spleen significantly. Data are represented as mean ±SEM. (C) Iguratimod decreases abnormal B220+T cell population of MRL/lpr mice. Data are represented as mean ±SEM. (D) Iguratimod decreases plasma cell population (CD138+) in spleen. Data are represented as mean ±SEM. (E) Representative kidney sections stained with CD20 after 20-week treatment of iguratimod or vehicle solution. B cell infiltrates in glomerulus and interstitial of kidneys from control mice. (F) Semiquantitative IOD values of all samples are analyzed. Each dot represents mean IOD value of an individual mouse. The horizontal lines represent media. Statistics are calculated by non-paired student's t test (B, C and D) or Mann-Whitney U test (F). * *p*<0.05, *** *p*<0.001.

Due to impaired apoptosis of lymphocytes, abnormal B220+ T cells accumulate in the periphery with autoimmune progression and dilute the proportion of normal B =  and T cells in MRL/lpr mice. [15], [18], [19] We found that the splenic B220+ T cell population shrunk remarkably following iguratimod treatment, leading to increased total B cell proportion. Cyc also showed a similar decrease in the B220+ T cell population and a moderate increase in the B cell proportion (Fig. 3A–C), which is consistent with previous reports. [20] Moreover, we found that the population of plasma cells was decreased by approximately 50% following iguratimod or Cyc treatment, compared with the vehicle control group. (Fig. 3D) As the terminally differentiated antibody producer cell type, this decrease in the plasma cell population was consistent with our previous observations relating to serum immunoglobulin and autoantibody levels.

Further study on lymphocyte apoptosis showed that iguratimod had no obvious capability to induce apoptosis compared with the effects of Cyc *in vivo*. (Fig S4) This may explain observation that iguratimod did not decrease the total lymphocyte number to the level induced by Cyc. Our data suggested that iguratimod reduces B220+T cell and plasma cell populations via a non-cytotoxic mechanism.

Despite increased numbers in the periphery, B cell infiltration of target tissue was inhibited by iguratimod. CD20 staining of kidney sections showed less CD20+B cell infiltration of both the glomeruli and interstitial space in iguratimod-treated mice compared with the vehicle controls.(Fig 3E) (IOD median: 42.39, QR 3.581–135.4 vs. median: 555.3, QR 163.1–945.5, *p*<0.0001) (Fig. 3F).

### Iguratimod decreased production of BAFF, IL-6, IL-17A and IL-21

**Figure 4:**
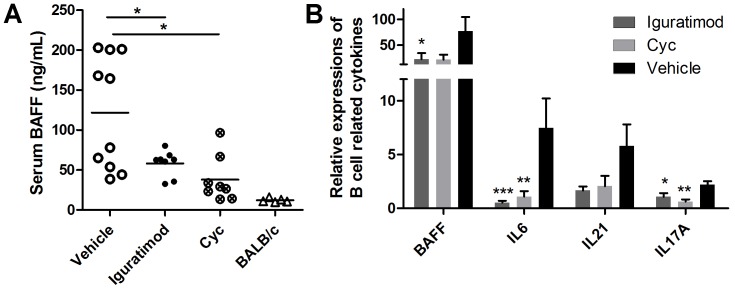
Iguratimod decreased production of BAFF, IL-6, IL-17A and IL-21. (A) Serum BAFF concentrations of the same mice above. Each dot represents an individual mouse. The horizontal bars represent mean. (B) Relative mRNA expression levels of BAFF, IL-6, IL-21 and IL-17A in splenic cells. The mice received 8-week treatment of iguratimod, cyclophosphamide or vehicle solution. Data are represented as mean ±SEM. Statistics are calculated by non-paired student's t test (A) or two-tailed Mann-Whitney U test (B). * *p*<0.05, **p<0.01, *** *p*<0.001.

Cytokines, including B cell activating factor (BAFF), interleukin (IL)-21, IL-6 and IL-17, are key players in the development of SLE [21] and are especially critical for B cell differentiation and plasma cell survival [22], [23]. Iguratimod significantly decreased serum BAFF concentration (Fig 4A), as well as mRNA expression in splenic cells of MRL/lpr mice. Similar decreases in the expression of IL-21, IL-6 and IL-17A in splenic cells were also observed in iguratimod-treated mice, with comparable effects to those of cyclophosphamide (Fig. 4B).

### Absence of overt toxicity

In previous clinical trials conducted by our group on RA, iguratimod showed no severe side-effects in RA patients at 50 mg/d. [3], [4], [24] The most common manifestations were reversible elevated liver enzymes and decreased peripheral white blood cell counts [4], [24]. Hence, we monitored serum alanine transaminase (ALT) and complete blood cell counts (CBC) of MRL/lpr mice at 15, 19 and 27 weeks of age during iguratimod treatment. The dose administered to the mice in this study was approximately two to three times that administered to RA patients, according to the dose translation equation [25]. There were no significant differences between the treated and control mice either in ALT or CBC levels at any time during the study (Fig S5). Furthermore, the iguratimod-treated mice showed no signs of severe infections in a non-pathogen-free environment, this indicating that long-term treatment with iguratimod is not associated with severe immunosuppression.

## Discussion

SLE is a complicated and treatment-refractory autoimmune disease. Nephritis is the most common complication that may be fatal. In contrast to the high heterogeneity of the disease, current options for disease modifying drug are still limited [26] and new agents for lupus treatment are urgently needed. Here, we describe a novel small molecular weight immunomodulator, iguratimod, which relieves multiple manifestations of lupus-like disease in MRL/lpr mice, including immune nephritis, joint swelling and changes in serological biomarkers. Our findings in a lupus-prone mouse strain suggest the potential therapeutic role of this agent in human SLE.

As a small molecular weight agent, iguratimod showed inhibitory effects on both T cell and B cell functions in previous studies. We found that iguratimod induced T cell response skewing away from Th17 in a CIA model, [1] and Luo *et al.* further demonstrated the direct effect of iguratimod on IL-17 signaling. [7] In experimental autoimmune encephalomyelitis, iguratimod directly inhibited myelin basic protein-specific T cell activation and pro-inflammatory cytokine production. [27] Iguratimod also effectively decreased immunoglobulin production by human and mouse B cells and suppressed antibody class switching. [9] In this study, we found that iguratimod significantly reduced the number of B220+ “autoreactive” T cells and plasma cells in the spleen. This suppressive effect was sufficient to significantly reduce hypergammaglobulinemia and anti-dsDNA autoantibodies as major hallmarks of polyclonal autoimmunity in SLE.

We observed decreased B cell infiltration in kidney tissues following iguratimod treatment, which is also observed in several other effective treatments for lupus patients or mice. [28], [29] It can be speculated that this effect is the result of iguratimod-induced downregulation of BAFF, which seems to be critical for B cell renal infiltration in patients with lupus nephritis. [30] This phenotype is probably with clinical relevance, since B cell infiltration *in situ* is strongly correlated with severe nephritis and poor prognosis in lupus patients [30] Furthermore, elevated levels of the B cell chemoattractant CXCL13 in target tissue or in circulation is positively correlated with disease activation both in patients and mice. [29], [31] The function of the infiltrating B cells is not clear; however, several studies reporting antibody producing by local plasma cells in the kidney [32], [33] may shed some light on this issue.

We found that IL-6, BAFF, IL-17A and IL-21 were downregulated by iguratimod in this study. All these cytokines play essential pathogenic roles in autoreactive T cell or B cell activation in MRL/lpr mice, [34]–[37] as well as in lupus patients. [26]. Targeting these cytokines can effectively treat lupus-like disease in mouse models; therefore, inhibition of these cytokines may help to explain the therapeutic effect of iguratimod on nephritis as well as its cellular effects on autoreactive T cell and plasma cell populations. Moreover, IL-6 [23] and BAFF [22] are also essential for the survival of plasma cells, which is crucially dependent on a complex microenvironment – the so-called ‘plasma cell niches’. Hence, modulation of the microenvironment by iguratimod may also contribute to decreasing plasma cell population.

Compared to rheumatoid arthritis, fewer new medicines have been approved for the treatment of SLE in recent years. Our data revealed that iguratimod effectively ameliorates immune nephritis of MRL/lpr mice, as well as other lupus symptoms, with relatively low risk side-effects. Taken together, these findings indicate that investigations of the therapeutic efficacy of iguratimod in human SLE in well-designed clinical studies are warranted.

## Conclusions

Iguratimod ameliorated antibody-mediated nephritis in MRL/lpr mice. This agent modified the imbalance in peripheral B cell differentiation probably through a non-antiproliferative mechanism. Our data indicate a potential therapeutic role of iguratimod in lupus.

## Supporting Information

Figure S1 Serum creatinine concentrations from iguratimod treated and control mice. Each dot represents mean ±SEM at the time point. Statistics are calculated by non-paired student's t test* *p*<0.05.
(TIF)

Figure S2 Thickness of front foot of mice receiving iguratimod or cyclophosphamide treatment is reduced at age of 18 weeks. Data are represented as mean ±SEM. Statistics are calculated by non-paired student's t test. **p<0.01.
(TIF)

Figure S3 Total splenic lymphocyte number from each group of mice after 8-week treatment. Data are represented as mean ±SEM. Statistics are calculated by non-paired student's t test. * *p*<0.05, **p<0.01.
(TIF)

Figure S4 Iguratimod lacks remarkable impact on lymphocyte apoptosis in *vivo*. Left, gating strategy of annexin V/PI double staining. The staining is applied to splenic cells of mice treated in each group for 8 weeks, identifying apoptotic cells (annexin V+, PI-). Right, apoptotic splenic cell percentage of each mouse from different groups. The difference between iguratimod and vehicle groups is not statistically significant. Data are represented as mean ±SEM. Statistics are calculated by non-paired student's t test. * *p*<0.05.
(TIF)

Figure S5 No overt toxic effects of iguratimod were found. Serum ALT and peripheral blood cell counts of iguratimod and vehicle solution treated MRL/lpr mice and BABL/c mice without treatment. Each dot represents mean ±SEM at the time point. Statistics were calculated by non-paired student's *t* test.
(TIF)
