# Supplementary figures and images for: Prevention of Immune Nephritis by the Small Molecular Weight Immunomodulator Iguratimod in MRL/lpr Mice
Source: PLoS One. 2014 Oct 1;9(10):e108273. doi: 10.1371/journal.pone.0108273 (PMC4182720; doi:10.1371/journal.pone.0108273)

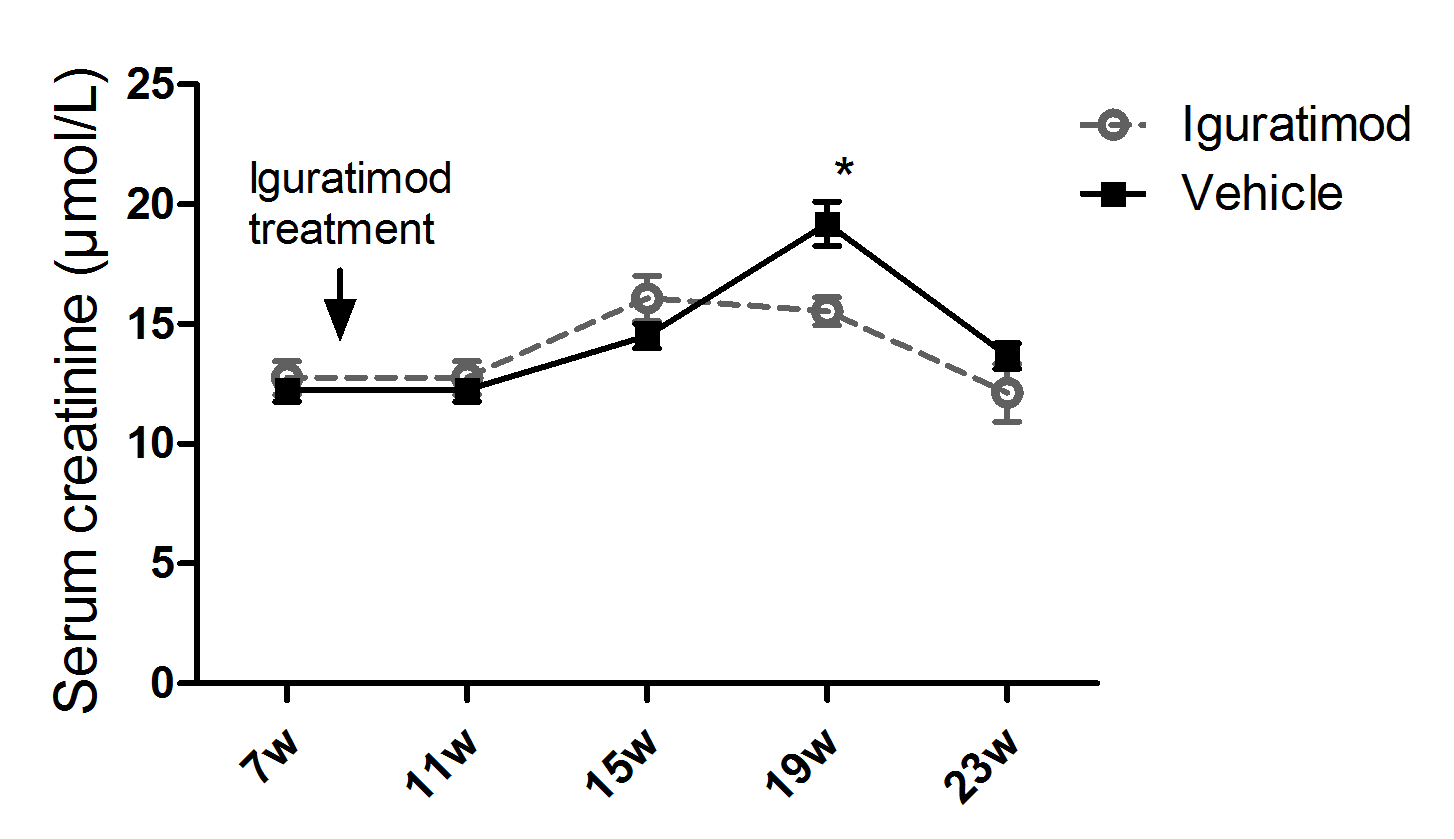

Supplement: Figure S1 — Serum creatinine concentrations from iguratimod treated and control mice. Each dot represents mean ±SEM at the time point. Statistics are calculated by non-paired student's t test* p<0.05. (TIF) [file pone.0108273.s001.tif]

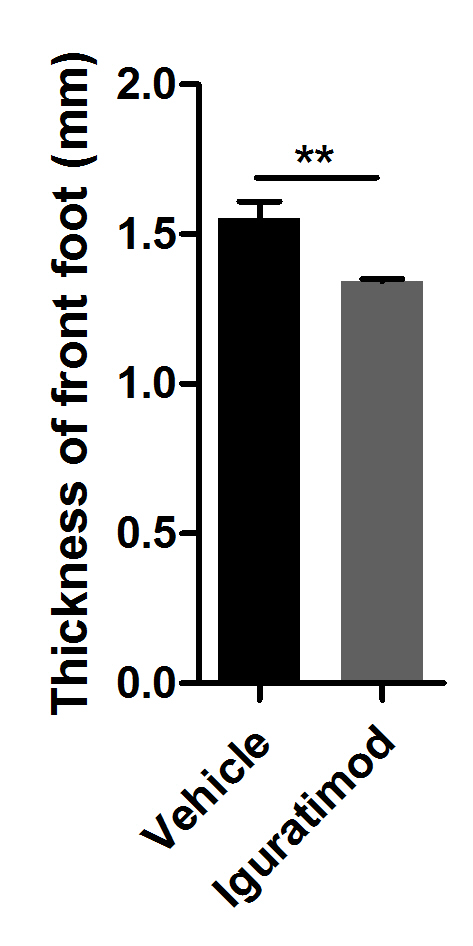

Supplement: Figure S2 — Thickness of front foot of mice receiving iguratimod or cyclophosphamide treatment is reduced at age of 18 weeks. Data are represented as mean ±SEM. Statistics are calculated by non-paired student's t test. **p<0.01. (TIF) [file pone.0108273.s002.tif]

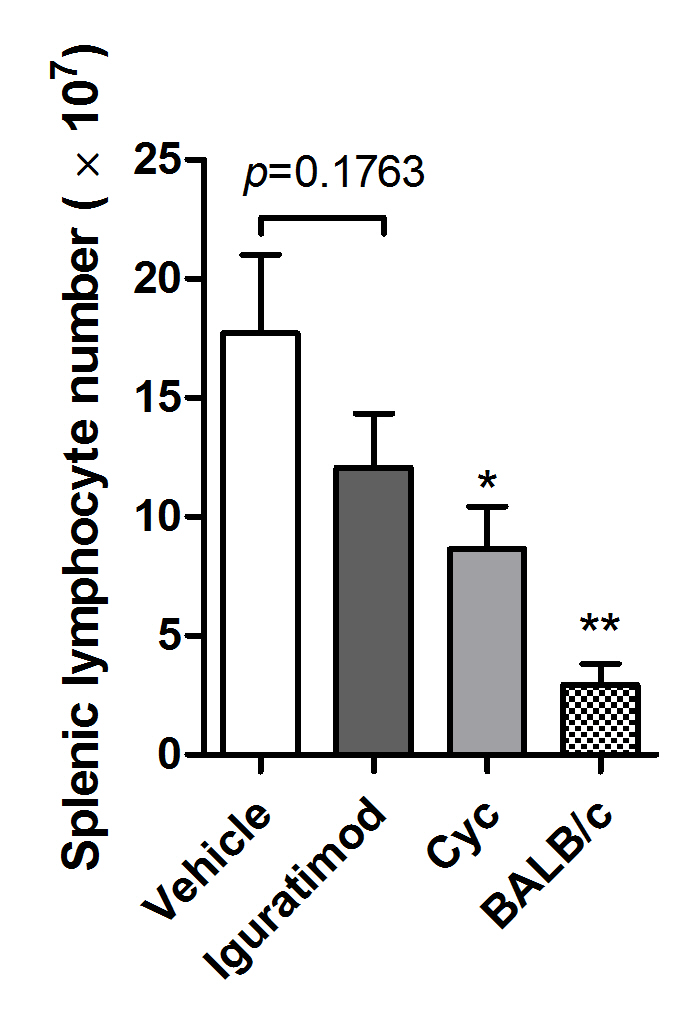

Supplement: Figure S3 — Total splenic lymphocyte number from each group of mice after 8-week treatment. Data are represented as mean ±SEM. Statistics are calculated by non-paired student's t test. * p<0.05, **p<0.01. (TIF) [file pone.0108273.s003.tif]

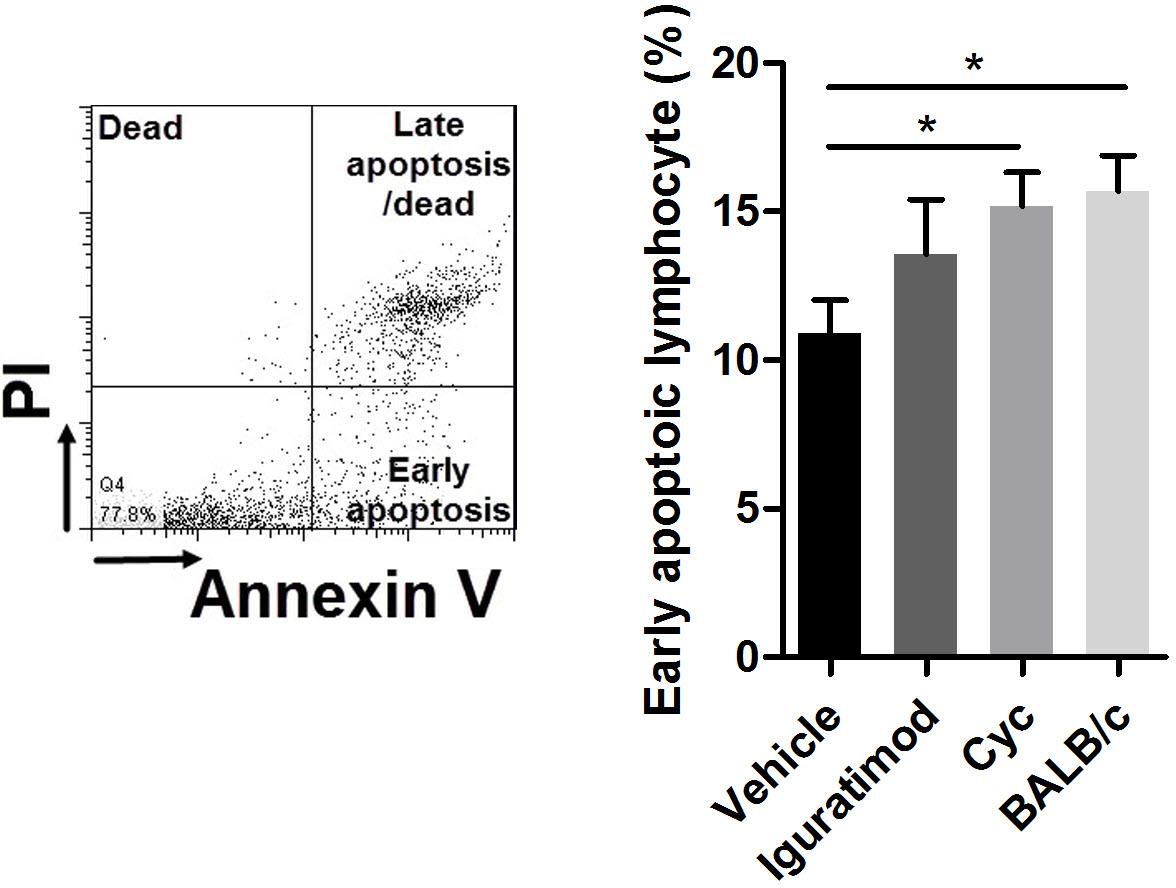

Supplement: Figure S4 — Iguratimod lacks remarkable impact on lymphocyte apoptosis in vivo. Left, gating strategy of annexin V/PI double staining. The staining is applied to splenic cells of mice treated in each group for 8 weeks, identifying apoptotic cells (annexin V+, PI-). Right, apoptotic splenic cell percentage of each mouse from different groups. The difference between iguratimod and vehicle groups is not statistically significant. Data are represented as mean ±SEM. Statistics are calculated by non-paired student's t test. * p<0.05. (TIF) [file pone.0108273.s004.tif]

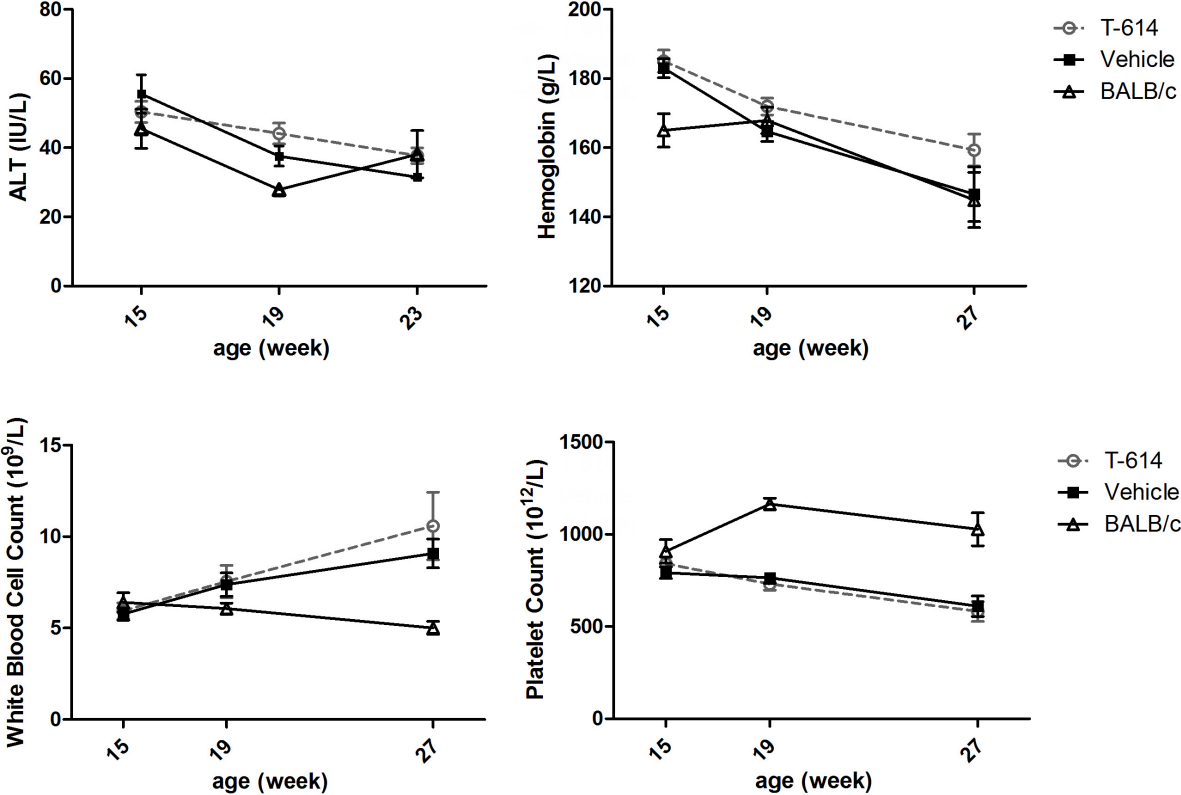

Supplement: Figure S5 — No overt toxic effects of iguratimod were found. Serum ALT and peripheral blood cell counts of iguratimod and vehicle solution treated MRL/lpr mice and BABL/c mice without treatment. Each dot represents mean ±SEM at the time point. Statistics were calculated by non-paired student's t test. (TIF) [file pone.0108273.s005.tif]
